# Response Comment on “Assessment of Ambient Air Pollution in Istanbul during 2003–2013”

**Published:** 2018-11

**Authors:** Eray YURTSEVEN, Suphi VEHİD, Merve BOSAT, Selçuk KÖKSAL, Cemile Nihal YURTSEVEN

**Affiliations:** 1. Public Health Department, Cerrahpasa Faculty of Medicine, Istanbul University, 34320 Kocamustafa Pasa, Istanbul, Turkey; 2. Health Management Department, Faculty of Health Sciences, Istanbul Kemerburgaz University, 34320 Sisli, Istanbul, Turkey; 3. Dept. of Sports Management, Faculty of Sport Sciences, Istanbul University, 34320 Avcılar, Istanbul, Turkey

## Dear Editor-in-Chief

Here we mention our responses to these valuable comments of M. Mahmoudimanesh.

1) We are sorry for the intendent use of Turkish syntax in paper. The maximum value of CO in 2003 is 3693 and its correct presentation is 3693.
2.) She may be right but this study should not be evaluated in biostatistics nor epidemiological aspects. It was is a descriptive study that we neither observed the epidemiological patterns of Asian and European sides of Istanbul nor we used advanced statistical techniques in order to forecast any outcomes depending on individual observations.

Best

**Eray YURTSEVEN**

Corresponding Author

